# Social implementation of a remote surgery system in Japan: a field experiment using a newly developed surgical robot via a commercial network

**DOI:** 10.1007/s00595-021-02384-5

**Published:** 2021-10-20

**Authors:** Hajime Morohashi, Kenichi Hakamada, Takahiro Kanno, Kenji Kawashima, Harue Akasaka, Yuma Ebihara, Eiji Oki, Satoshi Hirano, Masaki Mori

**Affiliations:** 1 Committee for Promotion of Remote Surgery Implementation, Japan Surgical Society, Tokyo, Japan; 2 Department of Gastroenterological Surgery, Hirosaki University Graduate School of Medicine, 5 Zaifu-Cho, Hirosaki, Aomori 036-8562 Japan; 3 RIVERFIELD Inc., Tokyo, Japan; 4 Department of Information Physics and Computing School of Information Science and Technology, The University of Tokyo, Tokyo, Japan; 5 Department of Gastroenterological Surgery II, Hokkaido University Faculty of Medicine, Sapporo, Japan; 6 Department of Surgery and Science, Kyushu University, Fukuoka, Japan; 7 Tokai University School of Medicine, Isehara, Japan

**Keywords:** Robotic surgery, Telesurgery, Glass-to-glass time, Communication delay

## Abstract

**Purpose:**

In recent years, the expectations for telesurgery have grown with the development of robot-assisted surgical technology and advances in communication technology. To verify the feasibility of the social implementation of telesurgery, we evaluated the communication integrity, availability, and communication delay of robotic surgery by remote control under different communication conditions of commercial lines.

**Methods:**

A commercial line was used to connect hospitals 150 km apart. We had prepared guaranteed-type lines (1Gbps, 10Mbps, 5Mbps) and best effort-type lines. Two types of robotic teleoperations were performed, and we evaluated the round-trip time (RTT) of communication, packet loss, and glass-to-glass time.

**Results:**

The communication delay was 4 ms for the guaranteed-type line and 10 ms for the best effort-type line. Packet loss occurred on the 5 Mbps guaranteed-type line. The mean glass-to-glass time was 92 ms for the guaranteed-type line and 95 ms for the best effort-type line. There was no significant difference in the number of errors in the task according to the type of line or the bandwidth speed.

**Conclusions:**

The social implementation of telesurgery using the currently available commercial communication network is feasible.

## Introduction

In September, 2001, the world’s first remote surgical procedure was performed between New York and Strasbourg, about 7000 km apart, using the surgical robot ZEUS^®^ [1], 2. Subsequently, 22 operations were performed remotely from Hamilton, Canada, at a hospital in North Bay, about 400 km to the north. [3, 4]. Although all these surgeries were successful, the transatlantic connection used an expensive dedicated line, whereas the Canadian connection used an IP-VPN line, a special inter-hospital network developed by the government. Other telesurgery studies in social settings conducted in the early 2000s used dedicated lines, commercial lines, or the Internet, but these were inadequate in terms of communication stability, integrity, security, and economic efficiency [5, 6]. Consequently, the underdeveloped information and communication technology was a decisive factor that led to a long hiatus in telesurgery research [7].

The recent development of high-speed, high-capacity communication technology using optical fiber and 5G, as well as new surgical robots, is making remote surgery a reality [8]. Moreover, there are growing expectations for remote surgery and remote surgical support from doctors with advanced skills, especially in areas with limited medical resources. If remote surgery becomes possible, local residents will be relieved of the physical, emotional, and financial burden of traveling long distances to undergo surgery [3, 7]. In Japan, there is a serious shortage of surgeons, especially in rural areas, partly because there is limited specialized surgical training outside of major population bases, so telesurgery is expected to be used for training young doctors in regional hospitals [8].

Telesurgery has been trialed for a variety of operations as it combines robotic technology and network communication technology with operator skill. Since there are still many technical issues to be verified and many social, ethical, and economic issues to be addressed before telesurgery can be implemented on a large scale in society, telesurgery has not yet reached full-scale practical use, and the issues hindering it need to be resolved [7, 9]. The most important factor to consider for the social implementation of telesurgery is the integrity and stability of the communication sources. Communication delays and packet loss lead to image turbulence and instability in robot function, which is a major risk to safe surgery [5, 10–12]. Therefore, it is essential to calculate the necessary bandwidth for communication according to the information in the video and operation signals of the surgical robot.

The latency and integrity of communication and information processing between a core hospital and a regional satellite hospital in the general social environment have never been measured using commercial communication lines in Japan, and the potential for social implementation of telerobotic surgery has not been verified. Before telesurgery can be effectively implemented in Japan, it is imperative to calculate the minimum delay time that surgeons can detect, using existing Japanese communication lines, and the level of delay time at which surgery becomes unsafe. The purpose of this study is to investigate the effect of the communication environment and bandwidth on robot function using a surgical robot for the purpose of realizing telesurgery, which is currently under development in Japan.

## Materials and methods

### Network connections

**Fig. 1:**
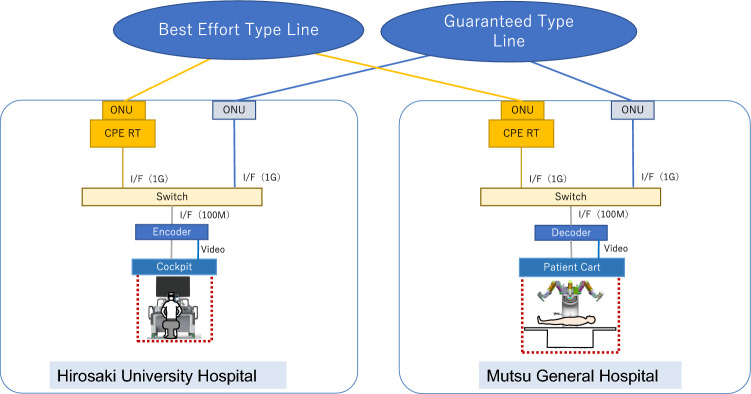
Network system. *OUN* optic network unit, *CPE RT* customer premises equipment remote terminal, *I/F* interface

Hirosaki University Hospital and Mutsu General Hospital in Mutsu City, 150 km north from Hirosaki City, were connected through a commercial fiber optic network. Two types of fiber optic networks were prepared: guaranteed-type lines (guaranteed bandwidth speeds of 1 Gbps, 10 Mbps, and 5 Mbps) and a best effort-type line (maximum speed of 1 Gbps) provided by the Nippon Telegraph and Telephone East Corporation (NTT East, Tokyo, Japan). An Internet Protocol–Virtual Private Network (IP–VPN) was also constructed using these lines. The guaranteed line can be adjusted according to the subscriber’s secured bandwidth usage needs. The service quality guarantee system and 24-h, 365-day support are standard features. The best effort line is a relatively inexpensive feature with a maximum transmission speed of 1 Gbps, with the actual speed varying, depending on the congestion of the line. Communication information is compressed and decompressed using Soliton's encoder: Zao-SH and decoder: Zao-View (Soliton Systems K.K., Tokyo, Japan) (Fig. 1). The encoders and decoders used in this study were Real-time Auto Speed Control based-on-Waterway model (RASCOW™), a high compression technology that enables ultra-short delay video transmission and has been applied to the ultra-short delay live broadcasting and remote control of construction equipment and automobiles. To evaluate the communication delay during the telerobot operation, we measured the round-trip time (RTT), which is the duration in milliseconds that it takes for a network request to go from the starting point to the destination and back to the starting point, the packet loss of image signals, and the glass-to-glass time, which is the combined transmission delay of five steps: (1) the laparoscopic camera delay; (2) the encoding delay; (3) the one-way delay in the communication line; (4) the decoding delay; and (5) the monitor response delay.

### Robot system and tasks

We used a surgical assist robot, which is being developed by Riverfield Inc. (Tokyo, Japan) [13].

#### (A) Relocation of bars

**Fig. 2:**
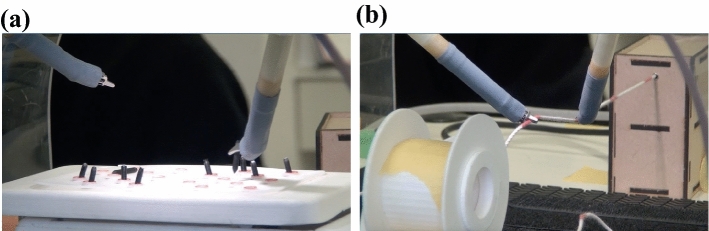
**a** Task ①: hauling on a rope Relocation of bars. **b** Task ②: relocation of bars

This task was designed to be one-handed (Task 1). The bars inserted into holes 1–10 on the left side of the table, which was mechanically shaken periodically, were moved to the holes numbered 1–5 on the right side. The bars were moved in numerical order. If a stick was dropped or inserted into the wrong numbered hole, it was counted as an error (Fig. 2a).

#### (B) Grasping and tugging a string

This task was designed to be two-handed (Task 2). Using a string with equally spaced markings, the left and right forceps were used to grasp the alternately marked areas, while the string was pulled in. Grasping the non-marked area or dropping the string were counted as errors (Fig. 2b).

The number of errors, along with the task completion time and the distance traveled by the forceps to complete each task, were measured. The subjects were six expert surgeons with extensive experience in robotic surgery. Three of the subjects had performed more than 100 robotic surgical procedures and three had performed about 30. There were also six non-expert surgeons with adequate experience in general surgery but no background in robotic surgery. Each task was performed three times. The tasks were performed in the order of 1 Gbps, 10 Mbps, 5 Mbps, and the best effort-type line.

## Results

### Communication delay and packet loss

#### (A) RTT

**Fig. 3:**
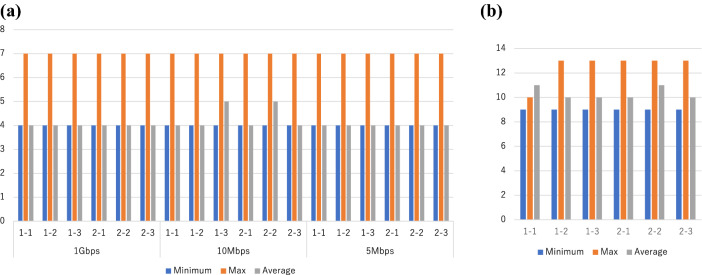
Example of a subject with delay during a task. **a** Guarantee type. **b** Best effort type. 1–1 represents the first measurement of Task 1. 1–2 represents the second measurement of Task 1; and 1–3 represents the third measurement of Task 1. 2–1 represents the first measurement of Task 2; 2–2 represents the second measurement of Task 2; and 2–3 represents the third measurement of Task 2

Figure 3 shows an example of the network communication delay during the task, where the mean RTT for the guaranteed-type lines (1 G, 10 Mbps, and 5 Mbps) was 4 ms (Fig. 3a). The mean RTT for the best effort-type line was 10 ms (Fig. 3b). Table 1 shows the results of the communication delay for all surgeons (*N* = 12). The mean RTT for the guaranteed-type lines was 4 (4–7) ms, and the mean RTT for the best effort-type line was 10 (9–13) ms (Table 1). For the best effort-type line, there was no difference in communication delay according to the time of day (Data not shown).

**Table 1:** Summary of all communication line delays

Line	Mean [min.–max.] (ms)
Guaranteed (1Gbps)	4 [4–7]
Guaranteed (10Mbps)	4 [4–7]
Guaranteed (5Mbps)	4 [4–7]
Best effort	10 [9–13]

#### (B) Packet loss

**Fig. 4:**
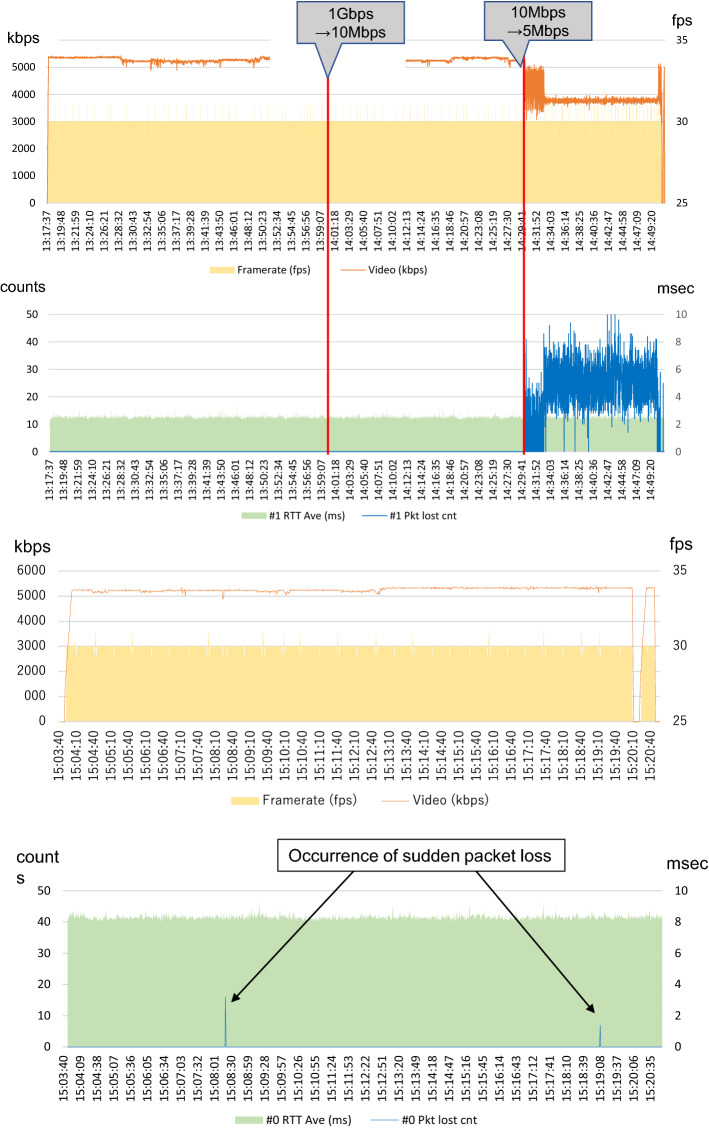
**a** Example of the typical video transmission frame rate, round trip time, and packet loss with the guaranteed type line. **b** Example of the typical video transmission frame rate, round trip time, and packet loss with the best effort type line. Framerate (fps): framerates per second. Video (kbps): video transmission value loss (kilobits per second). Pkt lost cnt: packet lost count

Figure 4 shows the video delivery value and the packet loss of the guaranteed-type line. There was no problem with the video transmission value or packet loss up to 10 Mbps; however, after switching to 5 Mbps, the video transmission value loss and packet loss were noticeable. (Fig. 4a). Although there was no delay on the 5 Mbps line, the image quality was degraded because of the packet loss. The best effort type line showed a slight but sudden packet loss that did not occur on the guaranteed-type line (Fig. 4b).

#### (C) Glass-to-glass time

The overall mean delay through the encoder–decoder, excluding network line delay, was 90 (70–110) ms. The mean delays of the endoscope camera processing and the monitor response accounts for 29 (22–36) ms, while the mean encoder–decoder delay itself was 61 (48–74) ms. Therefore, the glass-to-glass time was 92 ms for the guaranteed-type line and 95 ms for the best effort-type line (Table 2).

**Table 2:** Summary of the latency of information processing

	Mean [min.–max.] (ms)
Laparoscopic camera delay time	29 [22–36]
Encoder–Decoder delay time	61 [48–74]
Total delay (except communication line delay)	90 [70–110]
Glass to glass time (guaranteed line)	92 [74–114]
Glass to glass time (best effort line)	95 [80–120]

Glass-to-Glass time is defined as the total transmission time of the surgical field camera, the encoder, the communication line, the decoder, and the monitor

### Remote robot task

#### Error count

**Fig. 5:**
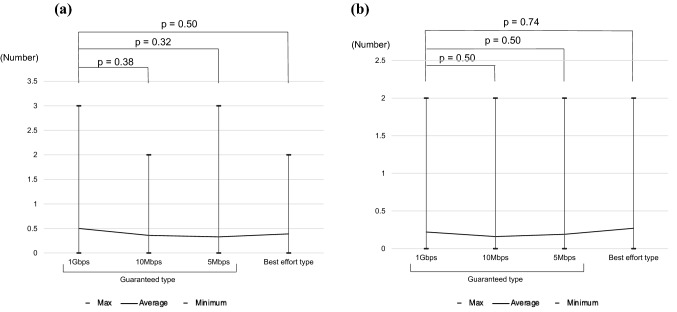
Number of errors of the two tasks. **a** Task 1. **b** Task 2

Figure 5 shows the number of errors for each task. The average number of errors for the guaranteed-type lines (1G, 10Mbps, and 5Mbps) and the best effort-type line in Task 1 was 0.5, 0.36, 0.33, and 0.39, respectively, with no significant difference (*p* = 0.50). For Task 2, the mean error counts were 0.22, 0.16, 0.19, and 0.27, with no significant difference (*p* = 0.74).

### Task completion time and total distance of forceps movement

**Fig. 6:**
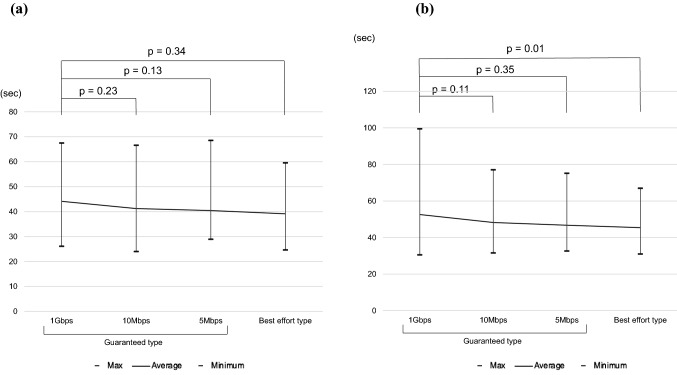
Completion time for the two tasks. **a** Task 1. **b** Task 2

Figure 6 shows the average task completion times for the guaranteed-type lines (1G, 10Mbps, 5Mbps) and the best effort-type line for the two tasks. The completion times for Task 1 was 44.1 s, 41.2 s, 40.4 s, and 39.1 s, with no significant difference by line type (*p* = 0.34). For Task 2, the times were 52.6 s initially, and then 48.2 s, 46.7 s, and 45.4 s, indicating that they became significantly shorter as the task progressed (p = 0.01).

**Fig. 7:**
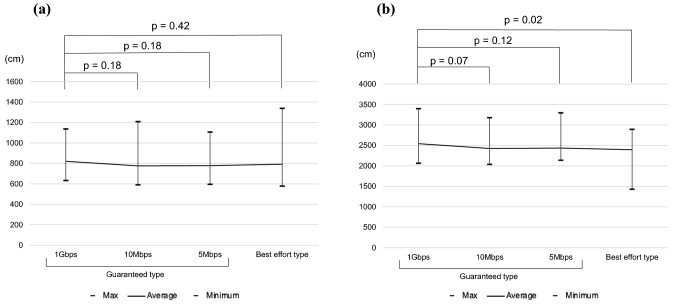
Total distance of forceps movement of the two tasks. **a** Task 1. **b** Task 2

Figure 7 shows the total distance of forceps movement in the two tasks. The mean forceps-movement distances for the guaranteed-type lines (1G, 10Mbps, 5Mbps) and for the best effort-type line in Task 1 were 821 cm, 776 cm, 777 cm, and 792 cm, respectively, with no significant difference by line type (*p* = 0.42). In Task 2, the distances were 2542 cm, first, and then 2425 cm, 2436 cm, and 2395 cm, indicating that they became significantly shorter as the task progressed (*p* = 0.02).

### Expert vs. non-expert outcomes

**Fig. 8:**
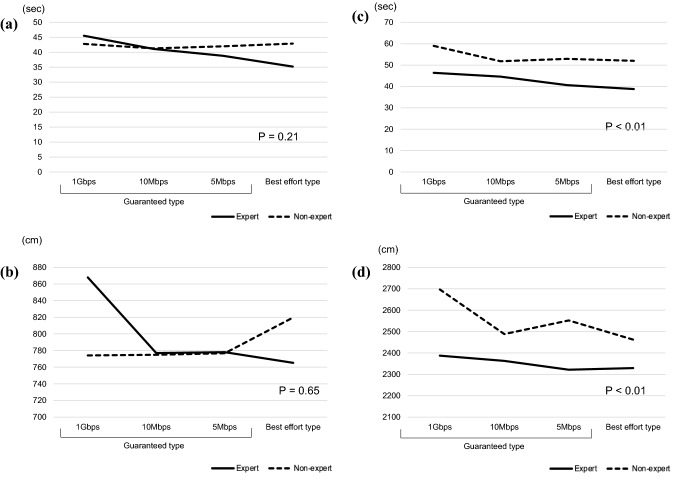
Comparison of task performance of experts vs. no-experts. **a** Time of Task 1. **b** Total movement of Task 2. **c** Time of Task 1. **d** Total movement of Task 2

In comparing the experts and non-experts, there was no significant difference in task completion time for Task 1 (*p* = 0.21), and no significant difference in forceps movement distance (*p* = 0.65). In Task 2, which was performed with both hands, for the experts, the task completion time was significantly shorter (*p* < 0.01) and the total forceps movement was also significantly less (*p* < 0.01). Moreover, the experts became faster as the task progressed, and comfort with the task led them to perform more strongly than the non-experts (Fig. 8).

## Discussion

The average communication delay was 4 ms for the guaranteed-type line and 10 ms for the best effort-type line, both representing very small delays. The glass-to-glass time for the guaranteed-type line was 92 ms, and the glass-to-glass time for the best effort-type line was 95 ms; much shorter than the values reported previously on communication delay, and the effect of delay seemed limited [5, 6, 10, 14]. The communication delay in this verification was considered a serviceable delay speed in many techniques; however, some surgeons commented that it was difficult to see the images on the 5 Mbps guaranteed-type line, and an examination of the experimental data revealed that packet loss occurred with the images. Although there was no delay in the 5 Mbps line, the image information was reduced by the late control function of the encoder, suggesting that the image quality decreased because of the packet loss. However, since the packet loss was retransmitted to the decoder by the retransmission processing function of the encoder, with a slight communication delay, it was thought that the image did not drop. On the other hand, in the best effort-type line, there was a small sudden packet loss in the data for everyone, thought to be unrecognizable to humans, and was so infrequent that it did not create a problem.

For the robot manipulation task, there was no significant difference in the number of errors, the task completion time, or the forceps travel distance between the 1 Gbps guaranteed-type line, the 10 Mbps line, the 5 Mbps line, and the best effort-type line. Although the 5 Mbps line did not cause delays that would affect the telerobot task, it did reduce the amount of image information, suggesting a decrease in image quality. Therefore, a line with a performance of at least 10 Mbps is recommended for the verification of this system.

The experienced delay time cannot be reduced to zero as the sum of the communication delay and the information processing delay [15]. Therefore, it is necessary for surgeons to calculate how long a delay time is acceptable for surgical execution. With regard to transmission delay, it has been reported that operability decreases when the delay time experienced by the surgeon exceeds 200 ms, that errors increase when the delay time exceeds 300 ms [3, 14, 16], and that tasks are almost impossible when the delay time exceeds 700 ms [17]. Many reports suggest keeping the delay time below 200 ms, ideally at 100 ms or less, to perform normal robotic operations [7, 18, 19]. The fifth-generation mobile communication system (5G) has the advantages of high-speed, high-capacity communication, high mobility, multiple connections, and wide bandwidth; beneficial for robots that require wider bandwidth for high quality transmission, such as 4 K/8 K images in the future. It is also expected to enable remote surgery in isolated areas, where it is difficult to lay wired Internet cables. Clinical cases and empirical research results of remote surgery using 5G have been reported [20, 21]. Techniques to reduce signal delays are improving, but several measures have been reported to enhance task efficiency and reduce the negative effects of the delays that inevitably remain. Orosco et al. reported the use of negative motion scaling to improve task handling for communication delays [22]. Xu et al. reported that 1 week of simple task training on robot manipulation caused by transmission delay is useful for complex tasks, indicating that manipulation from transmission delay can be improved by training [16]. In this study, we analyzed the phenomenon of operator comfort with a task, as well as the number of robot operations. Since the characteristics of the system affect the operator, it was necessary to evaluate each robotic system individually.

For remote surgery, it is important to select an appropriate communication network and secure sufficient communication bandwidth. There are two types of communication networks: open networks and closed networks, which differ in the degree of security assurance as well as in communication quality and expense. Information and communication processing technology that compresses and decompresses the transmitted data is also important during data transmission. Video signals account for the largest volume of transmission signals in telesurgery and are strongly affected by the communication bandwidth. Therefore, information compression processing technology is essential, but the process of compression and decompression also causes delays. Since there is a trade-off between the compression rate and the time required for compression and decompression, it is necessary to develop encoders and decoders that achieve high compression and low latency.

In telesurgery, it is important to select a communication network based on the premise of sufficient communication quality and communication security, while considering economics. In general, the more robust the network is, the more stable the communication and the more stable the robotic operation will be, but accordingly, the higher the communication cost will be. Since each surgical robot requires a different amount of bandwidth, it is necessary to select a closed-type line that can prioritize and secure a communication bandwidth that exceeds what is required. It is also necessary to prepare a backup line that can respond to communication interruptions to provide communication redundancy. It is necessary to discuss the selection of economical communication lines to find the most affordable solution that takes into account the characteristics of the robot. We confirmed that stable communication of robot motion signals was possible even with a best effort-type commercial line, owing to the intervention of an appropriate encoder and decoder.

It is expected that various robot systems, communication environments, and compression/decompression software will emerge as technology develops. Since the communication delay may differ depending on the combination of technology used in these systems, we must study many systems. Moreover, for the clinical application of telesurgery, it is advisable to prepare guidelines that organize the appropriate delivery system and focus on safety, ethics, and communication systems.

## Limitations

The entire safety zone was not assessed, because we did not measure the communication environment of 5–9 Mbps. Only measurements related to the delay of image signals were recorded, as measurements related to the delay of operation signals could not be taken. The communication distance was 150 km and no consideration was given to the communication delay and redundancy caused by multiple relay points. All surgeons were new to the Riverfield's robotic system and, therefore, were unfamiliar with it in the early stages of the tasks. Because of the limited time available for the experiment, there were only a small number of subjects who had 15 min of practice time just before the task. The order in which the tasks were performed also affected certain results, such as the number of errors and task completion time. Since the subjects did not perform the robot manipulation in a normal operating room, it was not possible to compare teleoperation with non-teleoperation.

## Conclusions

Under the communication environment of the telerobot operation system verified in this study, the communication delay was within a range that had little impact on the telesurgery. Thus, the social implementation of telesurgery using the currently available commercial communication network is feasible.
